# Investigating Racial Differences among Men in COVID-19 Diagnosis, and Related Psychosocial and Behavioral Factors: Data from the Michigan Men’s Health Event

**DOI:** 10.3390/ijerph18063284

**Published:** 2021-03-22

**Authors:** Jaclynn Hawkins, Karen Gilcher, Claudia Schwenzer, Michael Lutz

**Affiliations:** 1 School of Social Work, University of Michigan, Ann Arbor, MI 48109, USA; caschwen@umich.edu; 2 Michigan Institute of Urology Men’s Health Foundation, 419 Golf View Lane, Rochester, MI 48309, USA; gilcherk@miumenshealthfoundation.org (K.G.); lutzm@michiganurology.com (M.L.)

**Keywords:** COVID-19, men’s health, race

## Abstract

Extant research is growing in its ability to explain sex differences in novel coronavirus 2019 (COVID-19) diagnosis and mortality. Moving beyond comparisons based on biological sex is now warranted to capture a more nuanced picture of disparities in COVID-19 diagnosis and mortality specifically among men who are more likely to die of the illness. The objective of this study was to investigate racial disparities in COVID-19-related psychosocial, behavior and health variables among men. The present study utilizes a sample of 824 men who participated in a free health event held in a Midwestern state. Chi-square analysis showed that African American men were more likely to report an adverse impact of COVID-19 based on several factors including experiencing more COVID-19-related medical issues (χ^2^ = 4.60 *p* = 0.03); higher COVID-19 diagnosis (χ^2^ = 4.60 *p* = 0.02); trouble paying for food (χ^2^ = 8.47, *p* = 0.00), rent (χ^2^ = 12.26, *p* = 0.00), medication (χ^2^ = 7.10 *p* = 0.01) and utility bills (χ^2^ = 19.68, *p* = 0.00); higher fear of contracting COVID-19 (χ^2^ = 31.19, *p* = 0.00); and higher rates of death of close friends and family due to COVID (χ^2^ = 48.85, *p* = 0.00). Non-Hispanic white men reported more increased stress levels due to COVID-19 compared to African American men (χ^2^ = 10.21, *p* = 0.01). Regression analysis showed that race was a significant predictor of self-reported COVID-19 diagnosis (OR = 2.56, *p* < 0.05) after controlling for demographic characteristics. The results showed that compared to non-Hispanic White men, African American men were more likely to report an adverse impact of COVID-19 based on several factors including experiencing more COVID-19-related medical issues; higher COVID-19 diagnosis; trouble paying for food, rent, medication and utility bills; higher fear of contracting COVID-19; and higher rates of death of close friends and family due to COVID. Interestingly, non-Hispanic white men reported more increased stress levels due to COVID-19 compared to African American men.

## 1. Introduction

In the United States (US) and abroad, the novel coronavirus 2019 (COVID-19) has brought disparities in men’s health to the forefront of public health research [1]. Current available data shows that diagnosis rates among men and women are relatively similar, however, men are over twice as likely to die of COVID-19 than women [2,3]. For instance, one study utilizing a sample from China showed that men were more at risk for severity of symptoms and death from COVID-19 regardless of age [4]. Data also show that racial and ethnic minority groups are disproportionately represented among COVID-19 cases [4] which has important implications for men of color.

Griffith et al. (2020) brings to light the need to take a biopsychosocial approach to studying and developing solutions for men impacted by the COVID-19 pandemic [3]. While few in number, extant research shows that specific psychosocial and behavioral factors put men more at risk for adverse COVID-19 health outcomes including: lower adherence to social distancing and personal protective equipment (PPE) guidelines such as mask wearing and handwashing [5,6]. A study of Polish adolescents found that females were more likely to adhere to recommended protective behaviors compared to males, including hand-washing, social distancing, using gloves, and avoiding public places [7]. Further compounding the issue, attitudes and behaviors of men during the COVID-19 pandemic not only increase the threat of contracting and spreading COVID-19, but result in less treatment seeking until symptoms are severe [1,5,6,7]. For instance, recent studies have found that not only do men have less concerns about the pandemic, they also tend to have less knowledge of COVID-19 guidelines and health consequences compared to women [5,8].

On a national level, sex differences in COVID-19 diagnosis and death rates have been reported [2,4], yet a more nuanced examination of local level data is lacking to accurately explain and address gender disparities in COVID-19 and other illnesses on a community and individual level [3]. This is of particular importance given the local and state-level disparities in COVID-19 that exist in the U.S. [9]. For instance, in states such as Chicago, Louisiana and Michigan, while African Americans make up between 14% and 30% of the population, they account for a disproportionate number of COVID-19 cases and deaths, in some cases over 70% [9]. Further, discourses on gender equity in health primarily focus on a binary between men and women, which often ignore the structural and systemic issues that sub-groups of men and women are required to navigate [10,11,12,13,14]. This limited framing of gender equity does not acknowledge the complex health and social inequities faced by marginalized groups of men, particularly those relating to race, socioeconomic status, and geography [14,15].

Exploring psychosocial and behavioral factors related to COVID-19 that men engage in, while also considering the role of race and geography, are critical to better understanding gender-based disparities in COVID-19 diagnosis and mortality [3]. Such investigations also play an invaluable role in generating solutions to help men most at risk. Using a sample of men from the state of Michigan, the aim of the present study was to present a more nuanced picture of racial disparities in COVID-19-related psychosocial, behavioral and health variables and explores whether demographic and other variables can help explain racial disparities in diagnosis rates among men.

## 2. Materials and Methods

### 2.1. Sample

The present study utilizes a cross-sectional sample of 824 men who participated in a free health event in 2020. Participants were ages 18 or older, who could read and write in English, and who agreed to complete a paper-based self-reported survey given to each participant at the event. The free health event is hosted annually by the Michigan Men’s Health Foundation in Southeast Michigan. At the event, men are offered free screenings (valued at over $1800), including but not limited to prostate cancer screening, glucose, cholesterol, liver function and body fat percentage assessments, mental health screenings, HIV/AIDS testing, dental examinations, and more. The event also provides free flu shots, haircuts, guest speakers, and a free meal. In 2020, the Men’s Health Event provided 12,478 screenings to 880 attendees.

Volunteers and staff from the Michigan Men’s Health Foundation were trained to deliver an oral consent to participants and administered the survey to those who agreed to take it. Completion of the survey was voluntary, and participants were not offered any compensation. Items on the survey included Likert-type, multiple choice, and open-ended questions regarding help-seeking, family health history, personal health history, and a range of other biopsychosocial factors. The items included in this survey were developed by the Michigan Men’s Health Foundation in an effort to better meet the needs of low-income men in Southeast Michigan. While survey items were not validated, this dataset of survey responses was chosen due to ease of access to a population that has historically been under-sampled and under-studied in literature on health disparities [16].

### 2.2. Analysis

Descriptive statistics were used to describe the demographics of the sample. Between group chi-square analyses were conducted to determine if there were any significant differences in demographic, psychosocial, and behavioral factors between African American men and non-Hispanic white men in the sample. Regression analyses were used to determine whether racial differences in COVID-19 diagnosis persist, controlling for demographic and other factors and to determine which factors are associated with COVID-19 diagnosis. Cases with missing data were excluded from the analyses. Of the 880 participants in the 2020 sample, 6% were excluded for missing data, for a final sample of 824 for the current analyses. The analyses were conducted in SPSS Version 27.

### 2.3. Variables

Our primary outcome was race self-reported as African American and non-Hispanic white. COVID-19 diagnosis served as our primary dependent variable and was self-reported as “yes, tested positive for COVID-19” or “no, have never tested positive for COVID-19.”

### 2.4. Demographics

Education was coded “did not finish high school”, “high school/General Education Development Degree GED”, “some college” or “4 or more years of college”. Age was coded as a continuous variable and employment was measured by “yes employed” and “no unemployed.” Income was categorized as “<$35,000”, “$35,000–59,999”, and “>$60,000”, while marital status was coded as “single/widowed/separated/divorced” or “married/partnered/significant other”. Insurance type was coded as “no insurance”, “Medicare”, “Medicaid”, “private insurance”, and “veteran affairs insurance”. Last, health care utilization was coded as “yes” or “no” to the question “do you regularly see a doctor?”.

### 2.5. COVID-19 Psychosocial, Behavioral and Physical Health Variables

Psychosocial and behavioral related to COVID-19 variables were analyzed using 3 categories: (1) psychosocial and behavioral; (2) financial; and (3) physical health. Psychosocial and behavioral variables included sleep quality, stress level, experience with COVID-19 related death, comfortability with masks/social distancing, and fear of contracting COVID-19. Physical health related variables included delayed healthcare due to COVID-19, pain, heart issues, breathing issues and general medical issues. Lastly, financial variables were composed of housing stability, job loss, and ability to pay for rent and food (see Table 1).

## 3. Results

### 3.1. Demographics

Table 2 highlights the sample demographics. The majority of participants were self-identified as only African American (65%). Participants’ average age was 57 years, with most men reporting some college or four or more years of college (64% of African American men and 76% of white men). Fifty-four percent of African American men and 49% of white men reported an income of less than 35%, while 39% of white men had private insurance compared to 30% of African American men. It is important to note that the majority of African American and non-Hispanic white men in the sample had either no insurance, Medicare, or Medicaid, regardless of race (see Table 2).

### 3.2. Psychosocial and Behavioral Variables

For the purposes of this study, we categorized psychosocial and behavioral variables into three groups: COVID-19 Psychosocial Variables by Race/Ethnicity; COVID-19 Financial Difficulty Variables by Race/Ethnicity; and Physical Health Variables as a Result of COVID-19 by Race/Ethnicity. We describe our findings below:

#### 3.2.1. COVID-19 Psychosocial Variables by Race/Ethnicity (*N* = 824)

Table 3 shows the breakdown of psychosocial variables by racial group. Between racial groups, more non-Hispanic white men reported increased stress than African American men. African American men were significantly more likely to have experienced the death of someone close to them as a result of COVID-19 compared to non-Hispanic white men. Lastly, it was more common for African American men to report that they were “very afraid” of getting COVID-19 on a scale of 1–5 than non-Hispanic white men. For all other psychosocial factors, there were no statistically significant differences between the two racial groups.

#### 3.2.2. Covid-19 Financial Difficulty Variables by Race/Ethnicity

In addition to psychosocial factors, other variables relevant to COVID-19 were analyzed. Table 4 examines a range of issues that gauge the impact of the pandemic on men financially, highlighting any significant racial differences. African American men were more likely to report trouble paying for food, rent, medication, and utility bills than non-Hispanic white men in the sample. All other financial difficulty-related variables showed no significant differences between African American and non-Hispanic white men in the sample.

#### 3.2.3. Physical Health Variables as a Result of COVID-19 by Race/Ethnicity

Only two factors related to physical health impact during COVID-19 were found to have significant racial differences for men. Not surprisingly African American men were more likely to self-report a positive COVID-19 test than non-Hispanic white men. Lastly, African American men experienced more COVID-19-related medical issues than non-Hispanic white men in the sample. All other physical health variables showed no significant differences between African American and non-Hispanic white men in the sample (See Table 5).

#### 3.2.4. Logistic Regression for Racial Differences in COVID-19 Diagnosis

In the regression analysis, demographic variables noted in Table 1 were controlled for to determine racial differences in the outcome (COVID-19 diagnosis). While a range of psychosocial and behavioral factors related to COVID-19 demonstrated a significant racial difference between men and may drive racial differences in COVID-19 diagnosis, due to missing data and sample size, they were excluded from regression analysis for this study. Analysis showed that race was a significant predictor of self-reported COVID-19 diagnosis (*p* < 0.05). One way to interpret clinically meaningful findings is that the odds of getting a COVID-19 diagnosis is 156% greater for African American men compared to non-Hispanic white men. The model accounted for 6.4% of the variability in COVID-19 diagnosis (See Table 6).

## 4. Discussion

Racial disparities in COVID-19 diagnosis and a range of psychosocial and behavioral factors exist between African American and non-Hispanic white men residing in Michigan who took part in a free health event. At the onset of the pandemic, Michigan was and continues to be one of the hardest hit states by COVID-19. State level data shows that death rates attributed to COVID-19 are presently 15,854 [17]. While diagnosis rates are relatively the same between men and women, of this total more men have died as a result of contracting the illness; 6941 female deaths compared to 7941 male deaths were recorded [17], mirroring national level data of gender disparities in mortality [10]. Of these deaths, 1889 African American men have died from COVID-19 compared to 5603 non-Hispanic white men [17]. It is important to note, however, that African Americans only comprise 15% of the general population in Michigan, whereas non-Hispanic whites make up 80% of residents in the state [18].

Extant research demonstrates that, across the nation, lower income and more densely populated communities tend to have the highest rates of cases and deaths from COVID-19, but paradoxically have received the least assistance and resources related to the pandemic [4]. Our study shows that even among a group of low-income men utilizing a free health event, racial disparities in the impact of the pandemic persist. For example, analysis revealed that African American men were more likely to have COVID-19 and experienced more COVID-19-related medical issues compared to Non-Hispanic white men. African American men also reported higher rates of death of close friends and family due to COVID-19. These findings are consistent with current epidemiological data showing that African Americans in general are more likely to contract COVID-19, experience a higher severity of symptoms, and are more likely to die from the illness [19].

African American men also reported a greater negative impact of COVID-19 along a range of financial variables than their non-Hispanic white counterparts. These included experiencing more trouble paying for food, rent, medication, and utility bills as a direct result of COVID-19. Despite the fact that a majority of our sample were low-income men utilizing a free health event, racial disparities in the impact of COVID-19 on material resources persisted in our sample. This finding is not surprising considering African Americans and other racial and ethnic minorities in the US often have higher levels of medical comorbidities (i.e., diabetes, hypertension, obesity, and asthma) and lower socioeconomic status. Each of these factors can contribute to an increase in the risk of contracting COVID-19 through mechanisms such as weakened cell-mediated immunity to increased stress levels and lack of access to health care [19,20,21]. While the Centers for Disease Control (CDC) agrees that racial and ethnic background impacts susceptibility and mortality to COVID-19, the mechanisms through which these disparities persist remain largely unknown [22].

Further, by virtue of participating in a free health event, the finding that financial difficulties were encountered by African American attendees more than non-Hispanic white men was not surprising. For instance, the communities in which a majority of the African American sample population resides are low-income. In 2019, the median household income of Detroit households was $30,894 [18]. Additionally, over 30% of Detroit families live in poverty regardless of race and ethnicity [18]. While beyond the scope of our study, a closer examination of the neighborhoods in which men reside may be warranted. In general, African Americans are statistically more likely to live in communities with lower socioeconomic status. These neighborhoods are frequently characterized by a lack of access to quality medical care, grocery stores with healthy foods, substandard housing, and increased crime rates [19,20]. Indeed, research has shown that, when controlling for other demographic factors such as race, low socioeconomic status alone serves as a determinant of morbidity and mortality rates for a variety of illnesses [19,20,23,24]. Our findings point to the need for more rigorous research regarding how COVID-19 has created financial difficulties among African American men living in Michigan and whether or not this has impacted health outcomes.

Interestingly, non-Hispanic white men reported bigger increases in stress levels due to COVID-19 compared to African American men. Thus far, studies on the impact of COVID-19 have demonstrated that women report higher rates of stress and worse well-being compared to men [25,26]. To the authors’ knowledge, no studies have been conducted which examine racial disparities in stress and well-being due to COVID-19. Given the well documented impact of stress on engagement in healthy behaviors and susceptibility to disease [27,28,29], our study suggests that further investigation into this topic may be needed.

African American men expressed, more so than non-Hispanic men, an awareness of their increased risk for contracting COVID-19. Previous work by Gupta (2020) and others have found that men have engaged in more risk-taking behaviors and may be more likely to downplay the impact of the pandemic [30]. Given our finding that African American men experience more worry over contracting COVID-19, this may signal an opportunity to leverage these concerns via a public health prevention or treatment program.

Smith et al., (2020) provide a summary of strategies that have been employed to address a number of public health concerns taking into account the unique needs of men. These strategies include, but are not limited to: “settings- and place-based health promotion approaches, with a particular focus on engagement through faith-based settings, barber shops, sports clubs/organizations, and colleges and universities; involving peers and family; strengths-based approaches that focus on achievement, success and building leadership capacity; and approaches that address specific health issues, such as mental health and wellbeing and respective mechanisms associated with coping and resilience [31].” This existing program-related research, in addition to researchers, community organizers, health care professionals, and policy makers focused on men’s health, can and should be utilized to tackle pandemic related sex- and race-based disparities.

While surveys administered during a free health event such as ours are convenient and low cost, the limitations must be noted. The population studied was not geographically diverse, limiting the generalizability of our study on a national level. Further, it must also be noted that the majority of our sample was low-income. As a result, more analysis is needed with a larger more diverse sample to determine if racial differences persist across socioeconomic status and other biopsychosocial variables. Our study was also cross-sectional, and while we cannot track changes in COVID-19-related variables over time, the aim of our study was to generate local level data to paint a more precise picture of racial disparities in a range of factors among men. Lastly, while our survey items were not validated, their purpose was to gain a more nuanced picture of the needs of men at this event to develop more meaningful programming. In spite of these limitations, our study can potentially be used as a springboard for future work, with more rigorous survey items especially given the lack of data on low-income men and COVID-19.

## 5. Conclusions

The results showed that African American men were more likely to be diagnosed with COVID-19 compared to Non-Hispanic white men. Our study also found that on the whole, African American men were more likely to report an adverse impact of COVID-19 based on several factors including experiencing more COVID-19-related medical issues; trouble paying for food, rent, medication and utility bills; higher fear of contracting COVID-19; and higher rates of death of close friends and family due to COVID. Interestingly, non-Hispanic white men reported greater increases in stress levels due to COVID-19 compared to African American men.

Our study is unique and makes a significant contribution to the knowledge base because it is one of the only studies with a significant sample size of racially diverse men that asks specific questions regarding how the pandemic has impacted these men’s lives. A more concerted effort is necessary at a state and local level to redirect the pandemic response to those areas and populations hardest hit, namely low-income men, but more specifically African American men. The evidence-base also needs to expand to include a more nuanced picture of the individual, community, and state-level biopsychosocial factors the contribute to COVID-19 health disparities among men.

## Figures and Tables

**Table 1:** Psychosocial and behavioral variables.

As a result of Covid-19, did you experience trouble sleeping? Yes No As a result of Covid-19 has your stress level is Increased Same Less As a result of Covid-19, did you experience death of someone close to you? Yes No As a result of COVID-19, my overall quality of life is: Better Unchanged Worse Do you have any issues with wearing a mask or social distancing? Yes No How afraid are you of getting COVID-19? Scale of 1–5 1 (not afraid) 2 3 4 5 (very afraid) Have you delayed healthcare due to COVID-19? Yes No As a result of COVID-19, did you experience any legal issues? Yes No Has your pain increased as a result of the COVID crisis? Yes No How would you rate your daily pain during the past 6 months? 1 (no pain) 2 3 4 5 (high pain)	As a result of COVID-19, I’m having trouble paying for food. Yes No As a result of COVID-19, I’m having trouble paying my rent. Yes No As a result of COVID-19, I’m having trouble paying for medication. Yes No As a result of COVID-19, have you experienced job loss? Yes No Work hours reduced As a result of COVID-19, I’m having trouble paying my utility bills (gas, electric, water). Yes No As a result of COVID-19, did you experience loss of home/eviction? Yes No As a result of COVID-19, did you experience financial difficulty? Yes No Have you ever tested positive for COVID-19? Yes No As a result of COVID-19, did you experience any medical issues? Yes No As a result of COVID-19, did you experience breathing issues? Yes No As a result of COVID-19, did you experience heart issues? Yes No

**Table 2:** Main variable comparisons by race/ethnicity (*N* = 824).

Variable	African American (*n* = 539) *n* (%)	Non-Hispanic White (*n* = 285) *n* (%)	*p*
Education			0.00 *
Did not finish High School	37 (6.9)	11 (3.9)	
High School GED	157 (29.1)	57 (20.0)	
Some college	201 (37.3)	97 (34.0)	
4 or more years of college	144 (26.7)	120 (42.1)	
Age	57 (12.19)	58 (12.45)	0.28
Employment			0.43
Yes	258 (47.9)	139 (48.8)	
No	281 (52.1)	146 (51.2)	
Income ($)			0.00 *
<$35,000	291 (54.0)	140 (49.1)	
$35,000–59,999	164 (30.4)	72 (25.3)	
>$60,000	84 (15.6)	73 (25.6)	
Marital status			0.41
Single/Widowed/Separated/Divorced	259 (48.1)	140 (49.1)	
Married/Partnered/Significant Other	280 (51.9)	145 (50.9)	
Insurance Type			0.00 *
No insurance	119 (22.1)	77 (27.0)	
Medicare	151 (28.0)	68 (23.9)	
Medicaid	88 (16.3)	20 (7.0)	
Private Insurance	163 (30.2)	112 (39.3)	
VA Insurance	18 (3.3)	8 (2.8)	
Do you regularly see a doctor?			0.22
Yes	345 (64.0)	174 (61.1)	
No	194 (36.0)	111 (38.9)	

* *p*-Value less than 0.05 (≤0.05) is statistically significant.

**Table 3:** COVID-19 psychosocial variables by race/ethnicity (*N* = 824).

	African American (*n* = 539) *n* (%)	Non-Hispanic White (*n* = 285) *n* (%)	*p*	χ^2^
As a result of COVID-19, did you experience trouble sleeping?			0.64	0.219
Yes	152 (28.2)	76 (26.7)		
No	387 (71.8)	209 (73.3)		
As a result of COVID-19, your stress level is			0.01 *	10.21
Increased	234 (43.4)	156 (54.7)		
Same	271 (50.3)	118 (41.4)		
Less	34 (6.3)	11 (3.9)		
As a result of COVID-19, did you experience death of someone close to you?			0.00 *	48.85
Yes	178 (33)	35 (12.3)		
No	361 (67.0)	250 (87.7)		
As a result of COVID-19, my overall quality of life is:			0.08	5.03
Better	24 (4.5)	9 (3.2)		
Unchanged	333 (61.8)	158 (55.4)		
Worse	182 (33.8)	118 (41.4)		
Do you have any issues with wearing a mask or social distancing?			0.15	2.06
Yes	36 (6.7)	27 (9.5)		
No	503 (93.3)	258 (90.5)		
How afraid are you of getting COVID-19? ‘Scale of 1–5			0.00 *	31.19
1 (not afraid)	82 (15.2)	59 (20.7)		
2	56 (10.4)	48 (16.8)		
3	146 (27.1)	89 (31.2)		
4	98 (18.2)	52 (18.2)		
5 (very afraid)	1157 (29.1)	37 (13.0)		
Have you delayed healthcare due to COVID-19?			0.47	0.023
Yes	192 (35.6)	100 (35.1)		
No	347 (64.4)	185 (64.9)		
As a result of COVID-19, did you experience any legal issues?			0.28	0.615
Yes	23 (4.3)	9 (3.2)		
No	516 (95.7)	276 (96.8)		

* *p*-Value less than 0.05 (≤0.05) is statistically significant.

**Table 4:** COVID-19 Financial Difficulty Variables by Race/Ethnicity (*N* = 824).

	African American (*n* = 539) *n* (%)	Non-Hispanic White (*n* = 285) *n* (%)	*p*	χ^2^
As a result of COVID-19, I’m having trouble paying for food.			0.00 *	8.47
Yes	119 (22.1)	39 (13.7)		
No	420 (77.9)	246 (86.3)		
As a result of COVID-19, I’m having trouble paying my rent.			0.00	12.26
Yes	106 (19.7)	29 (10.2)		
No	433 (80.3)	256 (89.8)		
As a result of COVID-19, I’m having trouble paying for medication.			0.01 *	7.10
Yes	78 (14.5)	23 (8.1)		
No	461 (85.5)	262 (91.9)		
As a result of COVID-19, have you experienced job loss?			0.13	4.05
Yes	136 (25.2)	62 (21.8)		
No	334 (62.0)	196 (68.8)		
Work hours reduced	69 (12.8)	27 (9.5)		
As a result of COVID-19, I’m having trouble paying my utility bills (gas, electric, water).			0.00 *	19.68
Yes	154 (28.6)	42 (14.7)		
No	385 (71.4)	243 (85.3)		
As a result of COVID-19, did you experience loss of home/eviction?			0.35	0.863
Yes	22 (4.1)	8 (2.8)		
No	517 (95.9)	277 (97.2)		
As a result of COVID-19, did you experience financial difficulty?			0.31	1.02
Yes	241 (44.7)	117 (41.1)		
No	298 (55.3)	168 (58.9)		

* *p*-Value less than 0.05 (≤0.05) is statistically significant.

**Table 5:** Physical and behavioral health variables as a result of COVID-19 by race/ethnicity (*N* = 824).

	African American (*n* = 539) *n* (%)	Non-Hispanic White (*n* = 285) *n* (%)	*p*	χ^2^
Have you ever tested positive for COVID-19?			0.02 *	4.60
Yes	31 (5.8)	7 (2.5)		
No	508 (94.2)	278 (97.5)		
As a result of COVID-19, did you experience any medical issues?			0.03 *	4.60
Yes	61 (11.3)	19 (6.7)		
No	478 (88.7)	266 (93.3)		
As a result of COVID-19, did you experience breathing issues?			0.29	0.508
Yes	33 (6.1)	14 (4.9)		
No	506 (93.9)	271 (95.1)		
As a result of COVID-19, did you experience heart issues?			0.90	0.015
Yes	14 (2.6)	7 (2.5)		
No	525 (97.4)	278 (97.5)		
Has your pain increased as a result of the COVID crisis?			0.46	0.53
Yes	66 (12.2)	40 (14.0)		
No	473 (87.8)	245 (86.0)		
How would you rate your daily pain during the past 6 months?			0.73	2.00
1 (no pain)	173 (32.1)	93 (32.6)		
2	126 (23.4)	66 (23.2)		
3	136 (25.2)	69 (24.2)		
4	73 (13.5)	34 (8.1)		
5 (high pain)	31 (5.8)	23 (8.1)		

* *p*-Value less than 0.05 (≤0.05) is statistically significant.

**Table 6:** Logistic regression for racial differences in COVID-19 diagnosis.

	B	S.E.	Sig. (*p*-Value)	Odds Ratio
Race				
African American	0.940	0.435	0.031 *	2.560
Insurance Status				
Uninsured	−0.849	0.542	0.117	0.428
Medicare	–0.202	0.482	0.675	0.817
Medicaid	−0.339	0.571	0.553	0.713
Veteran Affairs	−18.344	7699.383	0.998	0.000
Employment Status				
Unemployed	0.364	0.404	0.368	1.438
Marital Status				
Divorced/Single	−0.459	0.353	0.193	0.632
Education				
Did not finish high school	−0.943	1.064	0.375	0.389
High School graduate/GED	−0.454	0.502	0.366	0.635
Some college	0.197	0.392	0.616	1.217
Age (Yrs)	−0.009	0.016	0.556	0.991
Constant	−2.815	0.990	0.004	0.060

* *p*-Value less than 0.05 (≤0.05) is statistically significant.

## Data Availability

The data that support the findings of this study are available from the corresponding author (J.H.) upon reasonable request.
